# Trans-Arterial Chemoembolization Plus Systemic Treatments for Hepatocellular Carcinoma: An Update

**DOI:** 10.3390/jpm12111788

**Published:** 2022-10-29

**Authors:** Alessandro Rizzo, Angela Dalia Ricci, Giovanni Brandi

**Affiliations:** 1 Struttura Semplice Dipartimentale di Oncologia Medica per la Presa in Carico Globale del Paziente Oncologico “Don Tonino Bello”, I.R.C.C.S. Istituto Tumori “Giovanni Paolo II”, Viale Orazio Flacco 65, 70124 Bari, Italy; 2 Medical Oncology Unit, National Institute of Gastroenterology, “Saverio de Bellis” Research Hospital, 70013 Castellana Grotte, Italy; 3 Department of Specialized, Experimental and Diagnostic Medicine, University of Bologna, Via Giuseppe Massarenti 9, 40138 Bologna, Italy; 4 Division of Medical Oncology, IRCCS Azienda Ospedaliero-Universitaria di Bologna, Via Albertoni 15, 40138 Bologna, Italy

**Keywords:** hepatocellular carcinoma, systemic treatments, immunotherapy, locoregional therapies, TACE

## Abstract

Recent years have seen the advent of novel treatment options for hepatocellular carcinoma (HCC). Given a strong biological rationale supporting this strategy, multiple studies have explored the role of combination treatments including locoregional plus systemic therapies to produce a synergistic effect and enhance antitumor activity. Among locoregional therapies, several clinical trials assessing trans-arterial chemoembolization (TACE) have been recently presented and published. In the current paper, we discuss available evidence and current and future research on combined TACE and systemic treatments, including antiangiogenic agents, immune checkpoint inhibitors, and immune-based combinations for HCC patients.

## 1. Introduction

Hepatocellular carcinoma (HCC) accounts for approximately 75% of all primary liver tumors and is often diagnosed at an advanced stage, being associated with a high mortality rate and an aggressive clinical course [1,2]. In early-stage disease, liver transplantation (LT), radical surgical resection, and radiofrequency ablation (RFA) are commonly used [3,4]. However, most patients are not eligible for radical treatment and are treated with systemic or locoregional therapies [5,6]. Locoregional approaches include local ablative therapies and intra-arterial treatments: RFA is considered as the most frequently used ablative modality, and is the first-line option for non-surgical and non-transplant candidates with early-stage disease and tumor size smaller than 5 cm. Conversely, intra-arterial treatments encompass trans-arterial chemoembolization (TACE) and hepatic arterial infusion chemotherapy (HAIC), which allows for selective delivery of chemotherapeutic agents to the tumor [7].

TACE represents the standard approach for intermediate-stage disease without extrahepatic spread or vascular invasion [8]. TACE is commonly used by performing an injection of a chemotherapeutic agent, such as doxorubicin or cisplatin, followed by embolization, or by delivery of embolic drug-eluting beads (DEB) [9,10]. In recent years, important efforts have been conducted to assess the efficacy of combined systemic treatments plus locoregional therapies, such as TACE in this setting, in order to produce synergistic effects [11,12]. From a biological point of view, locoregional treatments have been suggested to upregulate vascular endothelial growth factor (VEGF) [13,14]; since VEGF has a crucial role in the pathogenesis of HCC, antiangiogenic agents have been tested in combination with locoregional therapies [15,16]. Similarly, immune checkpoint inhibitors (ICIs) have been investigated in combination with locoregional approaches to boost the antitumor efficacy of these therapies [17,18,19,20,21,22,23,24,25,26,27]. In the current study, we discuss available evidence and current and future research on combined TACE and systemic treatments in HCC patients.

## 2. TACE Plus Antiangiogenic Agents

Following the presentation and publication of practice-changing clinical trials assessing the role of antiangiogenic agents, the HCC medical community has focused its attention on the identification of strategies aimed at increasing the antitumor efficacy of antiangiogenic agents such as sorafenib [28,29]. For example, an interesting analysis from the SHARP phase III trial suggested that systemic treatment with sorafenib following TACE was associated with longer median overall survival (OS) and time to progression (TTP) compared with patients receiving placebo with prior TACE [30,31]. At the same time, it is worth noting that a large number of studies tried to investigate the efficacy of TACE plus sorafenib without reporting notable and practice-changing results. In the SPACE phase II trial, Lencioni and colleagues explored the role of adding DEB-TACE to “continuous” sorafenib or placebo for HCC patients without extrahepatic spread and macrovascular invasion, with multinodular disease and intermediate stage [32]. According to the study design, sorafenib (400 mg twice daily) was started one week prior to the first TACE; the primary endpoint was TTP by blinded central review, with time to macrovascular invasion/extrahepatic spread, OS, overall response rate (ORR), disease control rate (DCR), and safety also assessed as secondary outcome measures. An amount of 154 HCC patients received sorafenib and 153 patients received placebos, and no significant differences in terms of clinical outcomes were reported between the two groups [32]. In particular, median TTP in HCC patients treated with DEB-TACE plus sorafenib or DEB-TACE plus placebo was 169 and 166 days, respectively, with no differences in median OS and median time to macrovascular invasion/extrahepatic spread between the two groups [32]. The ORRs for patients in the sorafenib and placebo groups with post-baseline scans were 55.9% and 41.3%, respectively, and the DCRs were 89.2% and 76.1%, respectively. Similarly, the multicenter, randomized, placebo-controlled, phase III TACE 2 trial compared sorafenib plus DEB-TACE versus placebo—DEB-TACE in patients with liver-only HCC, Child-Pugh class A [33]. No statistically significant differences were observed in terms of progression-free survival (PFS) between the two groups [33]. Fatigue, abdominal pain, and diarrhea were the most frequent grade 3–4 adverse events (18% in sorafenib versus 13% in the control arm, 13% versus 8%, and 10% versus 3%, respectively); three deaths occurred in each arm that were attributed to DEB-TACE. In another phase III Asian study, the post-TACE trial, patients with Child-Pugh class A unresectable HCC and liver cirrhosis were randomized to sorafenib or placebo following at least 25% of tumor shrinkage in TACE [34]. The study failed to meet its primary endpoint of median TTP, since median TTP was 5.4 months and 3.7 months, respectively (*p* = 0.25) [34].

Another therapeutic strategy based on the interrupted use of sorafenib combined with TACE has been tested in the phase II SOCRATES trial, where sorafenib was interrupted only around TACE [35]. Eligibility criteria included histologically confirmed, unresectable disease beyond Milan criteria, ECOG-PS 0, 1, or 2, no extrahepatic spread, and Child-Pugh score equal or less than 8. According to the results of this study, TTP was 16.4 months, and median OS 20.1 months in patients with unresectable HCC receiving this combinatorial strategy. Disease control rate according to EASL criteria was 74.4%, and four patients (9%) became amenable to either radiofrequency ablation or liver transplantation. No unexpected toxicity was observed [35]. Similarly, the phase II START study investigated the role of sorafenib (interrupted only around TACE) plus TACE for HCC patients with unresectable disease [36]. Discontinuation related to adverse events was observed in 8.1% of cases, and only 4four cases of serious toxicities were due to sorafenib. More recently, a multicenter, randomized controlled trial, TACTICS, compared TACE plus sorafenib versus TACE alone in unresectable HCC patients [37]. According to the study design, the antiangiogenic agent was used prior to the first TACE, and primary endpoint was PFS; TACTICS met its primary endpoint, with a statistically significant benefit in PFS determined by sorafenib plus TACE compared with TACE alone (22.8 months versus 13.5 months, respectively) [37]. Moreover, the same study observed prolonged time to vascular invasion and time to extrahepatic spread in HCC patients receiving the combinatorial treatment [37]. Median OS was 36.2 months with TACE plus sorafenib and 30.8 months with TACE alone, while post-trial treatments with active procedures/agents were received by 47 (58.8%) patients in the TACE plus sorafenib group and 58 (76.3%) in the TACE alone group. Thus, in TACTICS trial, TACE plus sorafenib did not show significant OS benefit over TACE alone; however, clinical meaningful OS prolongation and significantly improved PFS was observed, something suggesting that the combination of TACE plus sorafenib may be considered a choice of treatment in intermediate-stage HCC, especially in patients with high tumor burden.

Sorafenib has not been the only antiangiogenic agent tested in combination with TACE. For example, the anti-VEGF antibody bevacizumab has been investigated in this setting [38]. In particular, this agent has been approved as part of the systemic treatment for several solid tumors, including non-small cell lung cancer, colorectal cancer, renal cell carcinoma, and ovarian cancer [39,40,41,42,43,44,45,46,47,48,49,50]. In addition, the combination of immunotherapy plus bevacizumab was tested in HCC patients; in particular, the practice-changing IMbrave150 assessing the combination of the anti-PD-L1 agent atezolizumab plus bevacizumab versus sorafenib monotherapy in previously untreated patients with advanced disease has marked a new era, with the results of this study leading to the approval of this first-line combination [51,52]. Bevacizumab was first evaluated in combination with TACE in a single-center pilot study [53]; according to the results of this trial, the addition of bevacizumab to TACE was associated with a statistically significant improvement in terms of PFS, with the combination treatment reporting an overall manageable safety profile [53]. In addition, the median OS was 61 months in the TACE plus observation arm and 49 months in the TACE–bevacizumab group. However, the subsequent AVATACE-1 trial exploring the role of TACE plus bevacizumab versus TACE alone was terminated early due to unacceptable toxicity reported in 32 HCC patients [54]. In an ongoing study, the DEMAND, the combination of atezolizumab plus bevacizumab is under assessment prior to or in combination with TACE for the treatment of HCC patients with intermediate-stage disease. In this randomized phase II trial, the primary endpoint if the 24-month survival rate, with ORR, PFS, safety, and quality of life also assessed as secondary outcome measures (NCT04224636).

Another antiangiogenic agent, lenvatinib, has been investigated in HCC patients in combination with TACE [55]. This multitarget tyrosine kinase inhibitor (TKI) reported prolonged survival in combination with TACE (n = 60) versus TACE alone (n = 60) in a retrospective Chinese trial [55]. In particular, the 1-year and 2-year OS rates were significantly higher for patients receiving TACE plus lenvatinib (88.4% and 79.8%, respectively) compared with TACE alone (79.2% and 49.2%). These findings were also confirmed in the PFS analysis and in terms of ORR (68.3% and 31.7%, respectively). The results of this study support the exploration of this therapeutic strategy in prospective clinical trials with large sample size. Lastly, the multitarget TKI axitinib has been investigated in combination with TACE in a phase II, single-arm study, reporting a 2-year OS rate of 43.7%, with a median OS of 18.8 months [56]. Among the evaluable population (44 patients), 40.9% (18 patients), and 27.3% (12 patients) achieved complete and partial responses, respectively. Common grade 3 or above axitinib-related complications included hand-foot skin reaction (14%) and hypertension (24%).

## 3. TACE Plus Immune Checkpoint Inhibitors

Immune checkpoint inhibitors (ICIs) including pembrolizumab, nivolumab, durvalumab, atezolizumab, and others, have been recently evaluated in HCC patients, and clinical trials assessing single-agent ICI have reported disappointing results [57,58,59,60]. Conversely, immune-based combinations have been more striking. Despite ICIs seeming to have finally found their role in HCC as part of combinatorial strategies, several questions remain unanswered. Among these, the lack of validated biomarkers of response represents an important issue since only a proportion of HCC patients benefit from immunotherapy [61,62]. ICIs have also been assessed and are currently under evaluation in combination with TACE. Firstly, a study conducted by Duffy and colleagues tested the cytotoxic T-lymphocyte-associated protein 4 (CTLA-4) antibody tremelimumab plus TACE in 32 BCLC stage B HCC patients [63]. Tremelimumab was administered at two dose levels (3.5 mg/kg and 10 mg/kg intravenously) every 4 weeks for 6 cycles; a confirmed partial response was reported in 26.3% of cases, and median TTP and OS 7.4 months and 12.3 months, respectively [63].

Several ongoing studies are assessing TACE plus ICIs. Among these, the addition of the PD-1 inhibitor nivolumab to TACE is under evaluation in a clinical trial (NCT03143270) where nivolumab is combined with DEB-TACE and the primary endpoint of the study is the number of participants with treatment-related adverse events. The trial has a planned enrollment of 20 HCC patients and the estimated primary completion date is in April 2024. The results of the phase II German IMMUTACE study (NCT03572582) have been recently presented at the ASCO Annual Meeting [64]. The trial investigated the efficacy and safety of nivolumab–TACE in multinodular, intermediate-stage disease, and recruited 59 patients at 10 German sites [64]. According to the study design, HCCs were treated with up to two TACE treatments followed by nivolumab (240 mg), which was started on day 2–3 following the first TACE and was continued until disease progression for up to two years. ORR was 71.4%, with partial response, stable disease, and complete response rate of 55.1%, 4.1%, and 16.3%, respectively [64]. At a median follow-up of 20 months, median PFS was 7.2 months and median time to subsequent systemic therapy of 24.9 months. Median OS in included patients was 28.3 months. Treatment-related toxicities of grade 3 or more were reported in 34.7% of patients. IMMUTACE provides evidence for the efficacy of TACE plus ICIs, and further studies aimed to better explore this topic are warranted. In the meantime, several similar trials are currently ongoing, including NCT03397654 and PETAL, which are exploring the sequential use of TACE and pembrolizumab. Similarly, the dual checkpoint blockade with nivolumab plus ipilimumab is under evaluation in another trial (NCT03937830).

## 4. Conclusions

Impressive steps forward have been recently made in the field of systemic therapies for advanced HCC, with novel treatments opening the doors of a new era. In parallel, combining new and old treatments with locoregional approaches, such as TACE, has been suggested to boost the antitumor activity. We have recently witnessed a growing interest in TACE plus systemic treatments combinations to enhance antitumor efficacy and to produce synergistic effects. Several unanswered questions remain: among these, the issue of whether predictors (including treatment-related adverse events, PD-L1 expression, tumor mutational burden, gut microbiome, etc.) could be used to properly select the right patients to receive locoregional therapies plus immunotherapy and to provide useful information for disease-monitoring as well as treatment-decision making remains of pivotal importance, and these combinatorial treatments represent a promising research avenue in this setting.

## Data Availability

Not applicable.
